# Development of clinically relevant in vivo metastasis models using human bone discs and breast cancer patient-derived xenografts

**DOI:** 10.1186/s13058-019-1220-2

**Published:** 2019-11-29

**Authors:** Diane Lefley, Faith Howard, Fawaz Arshad, Steven Bradbury, Hannah Brown, Claudia Tulotta, Rachel Eyre, Denis Alférez, J. Mark Wilkinson, Ingunn Holen, Robert B. Clarke, Penelope Ottewell

**Affiliations:** 1 Department of Oncology and Metabolism, Mellanby Centre for Bone Research, University of Sheffield, Beech Hill Road, Sheffield, S10 2RX UK; 2 Manchester Breast Centre, Oglesby Cancer Research Building, University of Manchester, Wilmslow Road, Manchester, M20 4GJ UK

**Keywords:** Breast cancer, Bone metastasis, ER+, ER−, PDX

## Abstract

**Background:**

Late-stage breast cancer preferentially metastasises to bone; despite advances in targeted therapies, this condition remains incurable. The lack of clinically relevant models for studying breast cancer metastasis to a human bone microenvironment has stunted the development of effective treatments for this condition. To address this problem, we have developed humanised mouse models in which breast cancer patient-derived xenografts (PDXs) metastasise to human bone implants with low variability and high frequency.

**Methods:**

To model the human bone environment, bone discs from femoral heads of patients undergoing hip replacement surgery were implanted subcutaneously into NOD/SCID mice. For metastasis studies, 7 patient-derived xenograft tumours (PDX: BB3RC32, ER+ PR+ HER2−; BB2RC08, ER+ PR+ ER2−; BB6RC37, ER− PR− HER2− and BB6RC39, ER+ PR+ HER2+), MDA-MB-231-luc2, T47D-luc2 or MCF7-Luc2 cells were injected into the 4th mammary ducts and metastases monitored by luciferase imaging and confirmed on histological sections. Bone integrity, viability and vascularisation were assessed by uCT, calcein uptake and histomorphometry. Expression profiling of genes/proteins during different stages of metastasis were assessed by whole genome Affymetrix array, real-time PCR and immunohistochemistry. Importance of IL-1 was confirmed following anakinra treatment.

**Results:**

Implantation of femoral bone provided a metabolically active, human-specific site for tumour cells to metastasise to. After 4 weeks, bone implants were re-vascularised and demonstrated active bone remodelling (as evidenced by the presence of osteoclasts, osteoblasts and calcein uptake). Restricting bone implants to the use of subchondral bone and introduction of cancer cells via intraductal injection maximised metastasis to human bone implants. MDA-MB-231 cells specifically metastasised to human bone (70% metastases) whereas T47D, MCF7, BB3RC32, BB2RC08, and BB6RC37 cells metastasised to both human bone and mouse bones. Importantly, human bone was the preferred metastatic site especially from ER+ PDX (100% metastasis human bone compared with 20–75% to mouse bone), whereas ER-ve PDX developed metastases in 20% of human and 20% of mouse bone. Breast cancer cells underwent a series of molecular changes as they progressed from primary tumours to bone metastasis including altered expression of IL-1B, IL-1R1, S100A4, *CTSK*, *SPP1* and *RANK.* Inhibiting IL-1B signalling significantly reduced bone metastasis.

**Conclusions:**

Our reliable and clinically relevant humanised mouse models provide significant advancements in modelling of breast cancer bone metastasis.

## Introduction

Bone metastasis in breast cancer is a significant clinical problem, with up to 80% of patients with late-stage disease developing secondary skeletal involvement. Current treatments for bone metastasis are palliative and do not provide life-prolonging benefit, and a high proportion of patients experience bone complications including pain, fracture, nerve compression and hypercalcaemia [1].

The development of new treatment strategies for this group of patients requires a better understanding of the molecular mechanisms behind metastasis to bone. It is widely accepted that metastatic lesions are formed by successive genetic and epigenetic changes in both tumour cells and the microenvironment, driving a process termed ‘the metastatic cascade’ [2]. Epithelial cells from the primary tumour undergo epithelial-mesenchymal transition (EMT). During this process, tumour cells acquire a migratory and invasive phenotype that allows them to invade locally through the surrounding stroma before entering the circulation directly or via the lymphatic’s. Dissemination through the circulation then requires adaptive changes for survival and evasion of immune responses, prior to extravasation into secondary sites. Secondary tumours occur by metastatic cell survival, proliferation and vascularisation to support the growing tumour foci [3].

Certain properties of metastatic cancer cells (e.g. migration and invasion) can be studied relatively quickly/cheaply using in vitro systems [4], but these models do not provide the tissue-specific context or reflect the multiple biological processes, cell types and molecular interactions involved. An ideal model should recapitulate as many steps of the metastatic cascade as possible, in order to facilitate the development of novel therapeutic strategies. Traditional experimental metastasis models involve the study of mouse tumours that colonised the skeleton [5, 6]; however, there are fundamental biological differences between humans and mice. Enhanced metabolic activity and longer telomeres in mice compared to humans are just two examples, which determine oncogenesis and translate to phenotypic differences [7]. These differences also include the development of cancer in mesenchymal tissues in mice, whereas human tumours arise mainly from epithelial cells [7]. Mouse mammary tumours are hormone-independent, requiring lower levels of oestrogen/progesterone to support their growth in comparison to the majority of human breast tumours that are the hormone responsive [8]. Mouse tumours are also significantly less likely to metastasise to bone compared with human tumours (reviewed in [7]).

Xenograft models whereby human cells/tissues are injected into immunodeficient mice are now routinely used. Whilst subcutaneous tumour implantation is convenient and allows the study of genetically manipulated cells or mice, it does not provide the optimal microenvironment for modelling metastatic disease, including spreading to bone. Direct orthotopic implantation of tumour cells into bone (most commonly the tibia) overcomes this problem by introducing a large number of tumour cells directly into bone, allowing the study of molecules involved in tumour growth at this site. However, intratibial injections are technically demanding and invasive, and the piercing of the cortex and displacement of the bone marrow induces a healing response, thereby changing local bone metabolism and hence the bone microenvironment [9]. It also circumvents the early steps of bone homing and invasion. The most commonly used bone metastasis model relies on intravascular injection of human cancer cells either via the left cardiac ventricle or the tail vein, with the success of the latter relying on specific bone homing cell lines [10, 11]. Although useful for investigating organ-specific extravasation, survival and proliferation, this method bypasses the early molecular cues that may be responsible for initiation of metastasis from the primary tumour. The use of bone metastatic cell lines selected by multiple in vivo passages of tumour cells isolated from bone metastases may also fail to replicate the changes that are associated with bone homing. These models are therefore considered models of organ colonisation and growth, rather than metastasis.

A more clinically relevant metastasis model may thus be provided by the use of orthotopic transplantation, where human breast cancer cells are implanted into murine mammary tissue and subsequent secondary tumour growth detected in a range of tissues. The ability to study all stages of the metastatic cascade is an advantage, and this model has been shown to more faithfully mimic human tumour histology, gene expression profiles and therapeutic responsiveness [12]. However, it does not represent organ-specific metastasis, and there is considerable variation in the frequency and site of secondary tumour growth. Current understanding of the metastatic process also proposes a role for the metastatic niche for the initiation of metastatic cells, as well as their homing and colonisation potential at secondary sites (reviewed in [13]). Species differences may therefore influence the ability of the primary tumour to prepare/initiate a future metastatic niche. In an attempt to develop more clinically relevant models, in which tumour cells metastasise to a metastatic tissue of human origin, researchers have developed “humanised” animal models whereby pieces of human bone are sub-cutaneously implanted into immunocompromised mice before injection of tumour cells. However, the few publications in which this model has been used to study breast [14, 15] and prostate cancer metastasis [16] have reported low tumour take rates in human bone by a number of cell lines injected via tail vein or via orthotopic implantation, with osteotropism seemingly limited to the SUM1315 breast cancer cell line [14].

In the current study, we have significantly refined the humanised bone metastasis model to generate the first patient-derived xenograft (PDX) models of human ER+ and triple-negative breast cancer metastasis to human bone implants. These models represent all aspects of the metastatic cascade, including tumour growth at the primary site, dissemination into the circulation, homing to the human bone environment and colonisation of human bone by disseminated tumour cells. Both ER+ and triple-negative models have a high propensity to metastasise to human bone implants, and the described methodology provides highly reproducible metastasis from human breast cancer cell lines and PDXs. Using these refined models, we investigated the molecular changes that breast cancer cells undergo as they progress through the different stages of the metastatic process, demonstrating the usefulness of these models in identifying novel genes/molecules that can be used as potential biomarkers or therapeutic targets for breast cancer bone metastasis.

## Materials and methods

### Animals

All experiments were carried out in 8–10-week-old female NOD/SCID or NOD/SCIDγ mice (Charles River, Kent, UK) maintained on a 12-h:12-h light/dark cycle with free access to food and water. Experiments were carried out in accordance with local guidelines and with home office approval under project licences 40/3531 and 70/08964 University of Sheffield, UK.

### Cell lines and patient-derived xenografts

Human breast cancer MDA-MB-231-Luc2-TdTomato (Calliper Life Sciences, Manchester UK), MDA-MB-231 (parental), MCF7 and T47D were originally purchased from European Collection of Authenticated Cell Cultures (ECACC) and stably transfected to express luciferase cultured in DMEM + 10% FCS (Gibco, Invitrogen, Paisley, UK) prior to injection [17]. Cell lines purchased from commercial sources have been authenticated in-house using short tandem repeat analysis of 10 loci. All cell lines were cultured in a humidified incubator under 5% C02 and used at low passage > 20.

A panel of patient-derived xenografts (PDXs) derived from breast cancer patients consisted of previously characterised BB6RC39 (ER+ PR+ HER2+); BB3RC31, BB3RC32, and BB2RC08 (ER+ PR+ HER2−); and BB6RC37 and BB6RC52 (ER− PR− HER2−) breast cancer xenografts that have not been cultured in vitro [18]. Data and metadata on PDX models are available in PDX Finder (http://doi.org/10/1093/nar/gky984, pdxfinder.org http://pdxfinder.org/) and the EuroPDX data portal (http://dataportal.europdx.eu). PDXs were frozen in 10% DMSO 90% FCS in liquid N_2_ for long-term storage before being revived by direct subcutaneous implantation of tumour pieces into female NOD/SCIDγ mice. PDXs were removed once tumours reached 1 cm^3^ and dissociated into a single-cell suspension using Miltenyi tumour dissociation buffer (130-095-929) under constant agitation for 90 min at 37 °C. PDXs were transduced to express TdTomato Luc2 via electroporation (using 2-μg plasmid per 2,000,000 live cells in nucleofectin buffer and Amaxa nucleofector system 1 (Lonza, Scientific laboratory Supplies, Nottingham)) before re-injecting 100,000 cells through the 4th nipples into the mammary gland (MIND model [19]) or left cardiac ventricle of mice.

### Surgical human bone tissue and implantation

Human femoral heads were collected with informed consent from postmenopausal women with rheumatoid arthritis undergoing routine total hip replacement surgery under HTA licence 12182, Sheffield Musculoskeletal Biobank, University of Sheffield, UK. Trabecular bone implants (0.5 cm^3^) were prepared from the femoral heads of patients undergoing hip replacement surgery. Briefly, femoral heads were clamped at the femoral neck into an Isomet 4000 Precision saw (Buehler, Coventry, UK). Horizontal 5-mm slices were cut using a Precision diamond wafering blade (Buehler) with the first slice comprising mainly cortical bone being discarded. Trabecular bone implants were excised from the bone slices using a bone trephine (5-mm diameter) before storing in sterile PBS at room temperature. Bone implants were subcutaneously positioned in the left and right flanks of NOD/SCID mice under isoflurane anaesthesia within 2–4 h of removal of the femoral head from the patient. Mice received a single subcutaneous analgesic injection (15 mg, Vetergesic, Alstoe Animal Health, York) immediately before surgery; plus broad spectrum antibiotic 10 mg/kg (Septrin, Aspen Pharma, Australia) was administered by sub-cutaneous injection immediately after bone implantation and 24 h later.

### Time course for bone implant characterisation

NOD/SCID mice (*n* = 20) were culled 7, 14, 21 and 28 days post human bone implantation (*n* = 5 per group, 2 implants/mouse) and tissue collected for downstream analyses. One bone implant was fixed in 4% PFA, one bone implant was snap frozen in liquid nitrogen and mouse spleen was harvested in ice-cold sterile PBS for immediate flow cytometric analysis of B cell populations.

### Administration of tumour cells and pharmacological agents to model bone metastasis

NOD/SCID mice (*n* = 10/group) underwent bone core implantation. Four weeks later 5 × 10^5^ MDA-MB-231-luc2-TdTomato, T47D or MCF7 cells or 1 × 10^5^ PDX cells were injected orthotopically or into the 4th mammary ducts in 33% matrigel: 66% PBS: 1% trypan blue, or 1 × 10^5^ PDX cells were injected via the left cardiac ventricle. Experiments were carried out 3 separate times using bone from different donors each time. To facilitate growth of ER+ tumour cells and PDXs, mice were supplemented with 12 mg/L 17β oestradiol (Sigma Aldrich, Poole, UK) via their drinking water starting immediately following injection of tumour cells. For pharmacological inhibition of IL-1R signalling, mice were administered 1 mg/kg/day anakinra or placebo (control) via subcutaneous injection (*n* = 10/group). Primary tumour growth and metastases were monitored weekly by IVIS imaging. Primary tumours from MCF7 or T47D cells were removed by surgical resection once they reached 1 cm^3^ and metastasis left to develop for 8 weeks. For MDA-MB-231-luc2-TdTomato and PDXs, mice were culled when primary tumours reached 1 cm^3^ or mice lost ≥ 10% of their body weight. Samples taken for Affymetrix gene expression analysis were considered to contain metastatic cells if a positive signal for metastasis was detected by IVIS. For all other samples and PDXs, metastases were further confirmed on histological sections by breast histopathologist Prof. Simon Cross (University of Sheffield).

### Multiphoton microscopy for cell viability and vascularisation

Cell viability and vascular permeability were assessed using multiphoton microscopy by the uptake and activation of calcein AM (Sigma Aldrich). Two mice per group received 30 mg/kg of calcein AM by intraperitoneal injection 24 h prior to culling. Bone cores from injected mice were frozen in OCT and kept at − 80 °C until analysis using a Zeiss 510 multiphoton microscope (Zeiss, UK) and LSM510 software (Zeiss, UK). Briefly, a × 20/0.8 lens and a chameleon laser were used for bone imaging. Calcein was detected using the Argon/488 laser and lectin using the 633 laser. The scan area utilised a 6 × 6 tile scan with an average Z-stack depth of 100 μm.

### Fluorescence-activated cell sorting (FACS) analysis for detection of human B cells in spleen

The spleen was harvested from each mouse and dissociated by mincing with a scalpel blade in ice-cold PBS. 1 × 10^6^ spleen cells were incubated with a cocktail of mouse-specific CD45-APC (1 μl, for the exclusion of mouse leukocytes), human-specific CD19-FITC (1 μl, 302205, BioLegend, for the detection of human B cells) and human-specific IgG-PE (1 μl, 409303, BioLegend). Analysis was performed using a FACSCalibur™ flow cytometer (BD Biosciences).

### Microcomputed tomography

Analysis of bone volume was carried out using a Skyscan 1172 X-ray–computed microtomograph (Skyscan, Aartselaar, Belgium) equipped with an X-ray tube (voltage, 49 kV; current, 200 mA) and a 0.5-mm aluminium filter. Pixel size was set to 7 μm. For each sample, cross-sectional images were reconstructed with NRecon software (version 1.4.3, Skyscan). Volume of interest was defined on the two-dimensional acquisition images by drawing a 4-mm circle. Trabecular bone volume fraction (BV/TV)—the ratio of the volume of bone present (BV) to the volume of the cancellous space (TV)—was calculated for 3 mm of the bone. Modelling and analysis were performed with the use of CTAn (version 1.5.0.2) and CTvol (version 1.9.4.1) software (Skyscan).

### Histology—detection of osteoclasts and osteocytes

Bone implants were fixed in 4% paraformaldehyde and analysed by uCT prior to decalcification in a solution of 1% paraformaldehyde/0.5% EDTA in PBS for 4 weeks, changing the solution at weekly intervals. Bone implants were embedded in paraffin wax from which 4-μm sections were cut using a microtome. Osteoclasts were identified by tartrate-resistant acid phosphatase (TRAP) staining. Briefly, dewaxed sections were incubated in acetate-tartrate buffer at 37 °C for 5 min followed by incubation in naphthol AS-BI phosphate, dimethylformamide in acetate-tartrate buffer for 30 min at 37 °C. Sections were placed in a solution containing sodium nitrite, pararosaniline and acetate-tartrate buffer for 15 min at 37 °C, before counterstaining with haematoxylin. Osteoclasts were quantified per millimetre of trabecular bone surface from whole sections using a ScanScope digital slide scanner and software (Aperio, CA, USA). In the same sections, the number of viable osteocytes was counted and expressed as a percentage of empty lacunae.

### Isolation of circulating tumour cells

Whole blood was centrifuged at 10,000*g* for 5 min, and the cell pellet re-suspended in 5 ml of FSM lysis solution (Sigma-Aldrich, Pool, UK) to lyse red blood cells. Remaining cells were re-pelleted, washed 3 times in PBS and re-suspended in a solution of PBS/10% FCS. Samples from 10 mice per group were pooled prior to isolation of TdTomato-positive tumour cells using a MoFlow High performance cell sorter (Beckman Coulter, Cambridge UK) with the 470 nM laser line from a Coherent I-90C tenable argon ion (Coherent, Santa Clara, CA). For gene expression analysis: TdTomato fluorescence was detected by a 555LP dichroic long pass and a 580/30 nm band pass filter. Acquisition and analysis of cells were performed using Summit 4.3 software. Following sorting, cells were immediately placed in RNA protect cell reagent (Ambion, Paisley, Renfrew, UK) and stored at − 80 °C before RNA extraction.

### Gene analysis

Total RNA was extracted using an RNeasy kit (Qiagen) and manufacturers’ instructions. For Affymetrix array analysis, RNA was amplified using an mRNA amplification kit (Ambion) before being reverse transcribed into cDNA using Superscript III (Invitrogen AB). cDNA from MDA-MB-231 cells grow in the mammary ducts that did not metastasise, MDA-MB-231 cells grow in the mammary ducts that did metastasise, their corresponding metastases in human bone implants and naïve human bone from the same patient were pooled into *n* = 3 per sample, and three sets of pooled samples were hybridised to separate whole genome Affymetrix arrays using Prime View Gene Expression Arrays (Applied Biosystems) (Raw data available via www.ncbi.nih.gov accession number GSE137842). Data were analysed using Qluocore software (www.Qluocore.com) and genetic pathways for functional annotation were constructed using DAVID functional annotation tools (https://david.ncifcrf.gov/tools.jsp). Confirmation of genes whose expression profiles were central to pathways identified as being associated with bone metastasis were validated using cDNA from MDA-MB-231, MCF7 and T47D cells grown in vitro*,* cells grown in mammary ducts that did not metastasise, cells that metastasised from mammary ducts, circulating tumour cells, their corresponding metastases in human bone implants and naïve human bone from the same patient on microfluidic plates (plate number: 2586577, Applied Biosystems). Expression profiles of genes associated with different stages of the breast cancer metastasis to bone were confirmed independently in three samples per group and in primary and human bone metastases from BB2RC08 and BB6RC37. Relative mRNA expression of *PRRT2* (Hs01005085_g1), *HRAS* (Hs00978050 g1), *FN1* (Hs01549976 m1), *SERPINE 1* (Hs01126606m1), *MAPK3* (Hs00385075m1), *CDC42* (Hs00918044_g1), *MYC* (Hs00153408_m1), *IL-1B* (Hs00174097_m1), *Caspase 1* (Hs00354836_m1), *AKT1* (Hs00178289_m1), *S100A4* (Hs00243202_m1), *CTSK* (Hs00166156_m1), *MAPK1* (Hs01046830_ml), *LAMB1* (Hs01055967_m1), *SPP1* (Hs00959010_m1), *IL1R1* (Hs00991010_m1), *BCL2* (Hs00608023_m1), *IKBKB* (Hs00233287_m1), *CLDN1* (Hs00221623_m1), *CDK2* (Hs01548894_m1), *BCL2L1* (Hs00236329_m1), *IL-1Ra* (Hs00893626_m1), *JUP* (Hs00233287_m1), *TNFRSF11A* (Hs00269492_m1) and *CTSK* (Hs00166156_m1) were compared with the housekeeping gene glyceraldehyde-3-phosphate dehydrogenase (*GAPDH*; Hs99999905_m1). To assess the expression profile of genes associated with either human or mouse origin bone and as osteoclast and osteoblast markers, RNA was reverse transcription using Superscript III (Invitrogen) before analysis using Taqman gene expression assays. Relative mRNA expression of *CTSK* (Hs00166156_m1 and Mm01255862_g1), *RANKL* (Hs00243522_m1 and Mm00437135_m1), *VEGF* (Hs00900054_m1 and Mm), *ACP5* (Hs00356261_m1), *ALPL* (Hs01029144_m1) and *OPG* (Hs00900358_m1) were compared with the housekeeping gene *GAPDH* (Hs99999905_m1 and Mm99999915_g1). Expression of ER/PR/HER2 were confirmed in PDX samples using Taqman gene expression assays for *ESRRG* (Hs00976243_m1), *PGR* (Hs01556702) and *ERBB2* (Hs01001580_m1) and CT compared with *GAPDH*. All PCR assays were performed using an ABI 7900 PCR System (Perkin Elmer, Foster City, CA) and Taqman universal master mix (all reagents were purchased from Applied Biosystems via Thermofisher, UK). Fold change in gene expression between treatment groups was assessed by directly inserting CT values into Data Assist V3.01 software (Applied Biosystems), and changes in gene expression were only analysed for genes with a CT value of ≤ 25.

### Immunohistochemistry

The presence of osteoblasts was detected by immunohistochemistry using an antibody specific for human osteocalcin (M184, Takara Bio Inc., Japan). To determine the origin of blood vessels in the re-vascularised bone implants, a dual immunofluorescence protocol using species-specific CD31 antibodies was performed to detect endothelial cells; human-specific CD31 (1:400, ab76533, Abcam, UK) and mouse-specific CD31 (1:400, ab56299, Abcam, UK). To establish whether genetic alterations observed between PDXs growing in the fat pad and those that had metastasised to bone were translated into changes in protein expression immunohistochemistry for IL-1B (1:200, ab2105, Abcam), IL1R1 (1:200, ab154524, Abcam), S100A4 (1:500, ab40722, Abcam, hRAS (1:200, ab97488, Abcam), DKK (1:200, Ab38594, Abcam), Gamma Catenin (1:25, 2309, Cell signalling), Fibronectin (1:50, ab32419, Abcam). Staining was visualised with corresponding biotin-conjugated secondary antibodies (Vector Laboratories, 1:200) and either avidin FITC (mouse) or avidin TRITC (human) (Vector Laboratories).

### Statistical analysis

All data are expressed as mean ± SEM. Statistical significance was tested for two-tailed paired or unpaired *t* test as appropriate using Prism 7 software (GraphPad, La Jolla, CA, USA). Statistical significance was defined as *P* less than or equal to 0.05.

## Results

### Bone viability following xenotransplantation

**Fig. 1:**
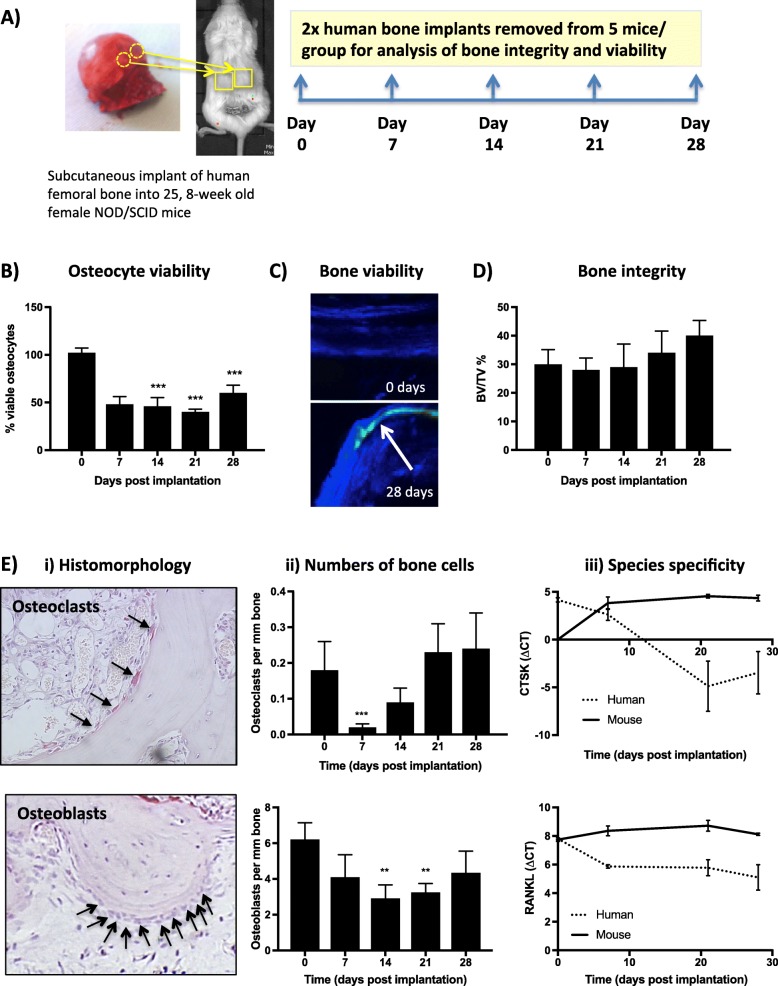
Viability of human bone xenograft following implant into NOD SCID mice. Two 0.5-cm^3^ pieces of human femoral head were implanted subcutaneously into 8-week-old female NOD SCID mice (*n* = 5/group). Animals were culled and bone implants removed 0, 7, 14, 21 and 28 days following implantation for analysis of viability (**a**). Percentage of viable osteocytes was assessed following Giemsa staining (**b**). Uptake of fluorescently labelled calcein by viable cells 0 and 28 days following implantation of bone was detected by multiphoton microscopy (**c**). Structure of bone was assessed by uCT and bone integrity shown as % bone volume compared with total tissue volume (BV/TV%) (**d**). Effects on osteoclast and osteoblast numbers and origin are shown in (**e**). (i) X60 photomicrographs of osteoclasts and osteoblasts lining human bone 28 days after implantation. (ii) Numbers of osteoclasts and osteoblasts per mm of bone surface, and (iii) origin of these cells as identified by expression of mouse and human CTSK (osteoclasts) and RANKL (osteoblasts/osteocytes) normalised to GAPDH. All graphs show mean ± SEM from 5 mice per time point. Data represents mean ± SEM with statistical significance determined by one-way ANOVA. *p < 0.01, **p < 0.001, ***p < 0.001 compared with 0 h control

To characterise the early stages of human trabecular bone engraftment following subcutaneous implantation in NOD/SCID mice, a time course was performed whereby cohorts of animals were culled at weekly intervals for 4 weeks following bone implantation (*n* = 5 per time point) (Fig. 1a). Bone cores were isolated and histological and gene expression analyses were performed to investigate bone viability and integrity.

Bone viability was investigated by quantification of osteocytes as well as functional labelling of viable cells visualised by multiphoton microscopy of calcein uptake (Fig. 1b, c). Despite positive calcein labelling, viability of the human bone was reduced as detected by the loss of osteocytes. There was a 57% fall in viable osteocytes (measured by quantitation of empty lacunae) by day 7 compared to day 0 controls (*p* < 0.0001). This was maintained at all later time points and did not decrease further. These results support that there is a substantial loss of bone cell viability in the initial stages following in vivo implantation, likely in part due to the lack of vascularisation of the bone cores and damage to the tissue during preparation of the bone implants. Nevertheless, the integrity of the bone cores was maintained throughout the time course experiment. There was no significant difference between the bone volume/tissue volume ratios of engrafted bone cores at any time point compared to day 0 baseline controls (Fig. 1d).

### Detection of functional bone cells in the bone cores

Bone forming osteoblasts and bone resorbing osteoclasts are considered to be components of the bone metastatic niche; we therefore assessed the presence and activity of these two key cell types using a combination of immunohistochemistry and gene expression. Osteoclasts on the bone core surfaces were clearly identified by TRAP staining together with their unique multi-nucleated morphology (Fig. 1e (i)). Following xenotransplantation, their number fell during the first week but steadily increased back to baseline control level by week 4 (NS, Fig. 1e (ii)). Species-specific gene expression of the osteoclast marker *CTSK* suggests that the majority of this recovery is attributed to repopulation by mouse-derived osteoclasts (Fig. 1e (iii)); however, functional human osteoclasts were still identified by detectable human CTX secretion in serum samples collected from 5 mice at day 28. Osteoblasts also remained present on the surface of human bones for the duration of the experiment (Fig. 1e (i)). Numbers of osteoblasts reduced 14 days after bone implantation (*p* < 0.001) but returned to normal levels by day 28 (Fig. 1e (ii)). This was due to increased recruitment of mouse osteoblasts; however, human osteoblasts remained present and could be detected by RANKL expression in bone for the 28-day time period of the experiment (Fig. 1e (iii)). These data indicate that by day 28, bone structure is normalised and is being actively turned over by both mouse and human osteoclasts and osteoblasts.

### Revascularisation and human B cell production following xenotransplantation

**Fig. 2:**
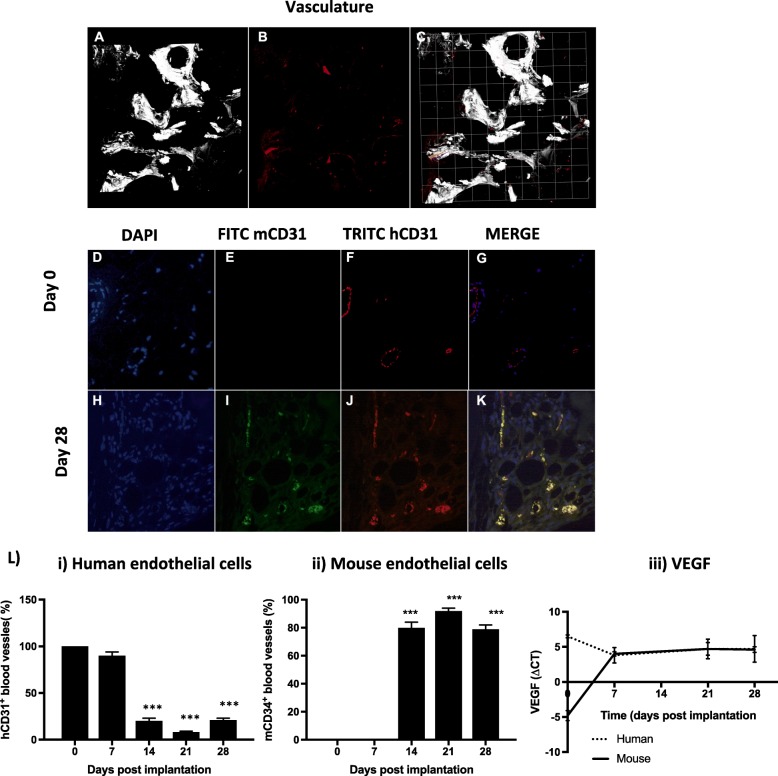
Vascularisation of human bone xenografts following implantation into NOD/SCID mice. Representative 3D reconstructions of bone architecture (**a**), tomato-conjugated lectin bound to carbohydrates on the luminal surfaces of blood vessels (**b**) and their merged location within the bone (**c**). Determination of species origin of endothelial cells is shown in panels **d**–**k**, representative immunofluorescent images of bone sections on day 0 (**d**–**g**) and day 28 (**h**–**k**). Single-channel DAPI (**d** and **h**), FITC-conjugated mouse CD31 (**e** and **i**), TRITC-conjugated human CD31 (**f** and **j**) and merged dual stained images (**g** and **k**). Total number of blood vessels present in 10 fields per bone was unchanged over the time course. Presence of single stained human blood vessels decreased significantly from day 14 (**l** (i)), but a concomitant increase in dual stained vessels was detected (**l** (ii)). Gene expression of human and mouse VEGF confirmed the immunofluorescent time curve (**l** (iii)). Data represents mean ± SEM with statistical significance determined by one-way ANOVA. ***p < 0.0001

Functional revascularisation of the bone core was confirmed by multiphoton microscopy of bone cores isolated from animals that had received an i.v. injection of Alexa-conjugated lectin prior to cull (Fig. 2a–c). To determine the species origin of endothelial cells responsible for vascular remodelling, dual immunofluorescence was performed on sections using both mouse- and human-specific CD31 antibodies. Day 0 controls show the presence of human CD31+ endothelial cells lining blood vessel walls with no cross-reactivity from the mouse CD31 antibody (Fig. 2d–k). The total number of CD31+ blood vessels was counted from 10 fields per bone core section for each time point. There was no loss in the number of vessels detected over time compared to day 0 controls with a mean range of 30–40 vessels per section. Vessels that stained positive for just human CD31 were present in bone sections for 7 days post implantation but fell significantly between day 14 and the end of the study (*p* < 0.0001). In contrast, vessels that stained positive for both human and mouse CD31 became evident from day 14 and comprised of 81% of all vessels (Fig. 2l (i, ii)), and these levels of dual stained vessels remained high (93% and 81%) at days 21 and 28 respectively. In contrast, no vessels staining exclusively for mouse CD31 were detected at any time point. To further establish the species derivation of the blood vessels, gene expression of VEGF in the implanted bone cores was quantified using human- and mouse-specific primers (Fig. 2l (iii)). The pattern of expression over the time course mirrored that of the immunofluorescent staining, with human VEGF expression decreased 1 week following implantation (mean ΔCT 5.7 ± 0.2 vs. 2.5 ± 0.5 at day 0 and day 7 respectively, *p* < 0.001). A concomitant increase in mouse VEGF was detected during the same time period (mean ΔCT − 4.2 ± 0.5 vs. 3.8 ± 0.4 at day 0 and day 7 respectively, *p* < 0.05). An equal level of murine and human VEGF expression was detected for the remainder of the study, suggesting that the vascularisation of the implanted bone discs is a consequence of angiogenesis and not vasculogenesis by the murine vasculature.

In addition, the presence of human B cells in the mouse spleen was investigated to support not only the ability of the bone core to re-vascularise but as evidence of the transfer of cells from one organ site to another, important for modelling metastasis.

**Fig. 3:**
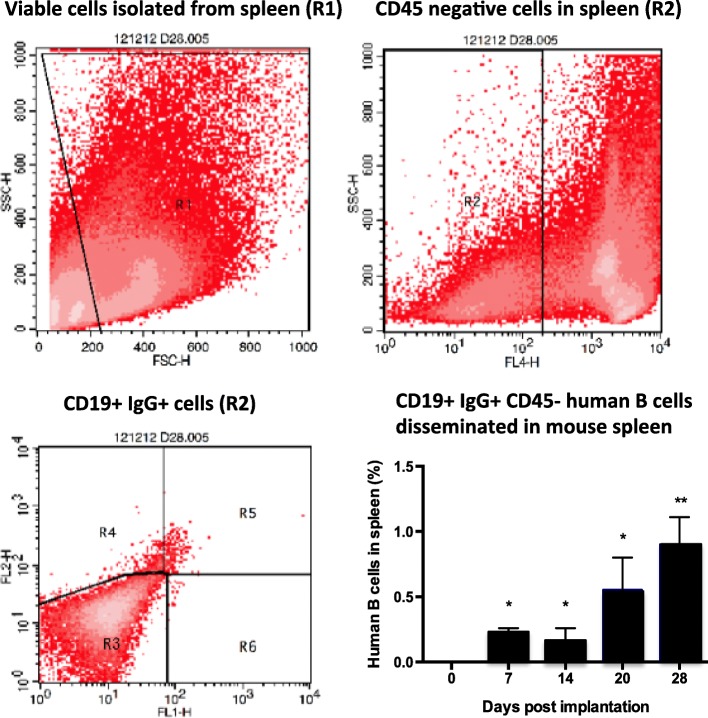
Evidence for functional activity of vasculature in bone implants. The spleen of NOD/SCID mice implanted with human bone was dissociated for the detection of human B cells disseminated (*n* = 5/group). Single-cell suspensions were labelled with antibodies specific for human IgG-PE and CD19-FITC and mouse CD45. FACS scatter plots were gated for all viable cells (R1), then mouse CD45-negative cells (R2). R5 from each spleen sample was plotted as a histogram for human CD19+ IgG+ cells for each time point. Data represents mean ± SEM, statistical significance determined by one-way ANOVA, *p < 0.01, **p < 0.001

Differentiation of B cells begins in the bone marrow and is completed in the spleen. Therefore, viability of the human bone cores and its bone marrow was first determined by the identification of human B cells in the mouse spleen (Fig. 3). Single-cell suspensions from the spleen were first separated by flow cytometry into a mouse CD45-negative population. From this population human B cells were identified by double-labelling for human CD19 and human IgG. The number of human B cells detected in spleen cell suspensions increased with time, representing 0.19, 0.13, 0.55 and 0.94% of the total number of cells at days 7, 14, 21 and 28 respectively, reaching significance by day 7 (*p* < 0.05).

### Metastasis of breast cancer cell lines to human bone implants

**Fig. 4:**
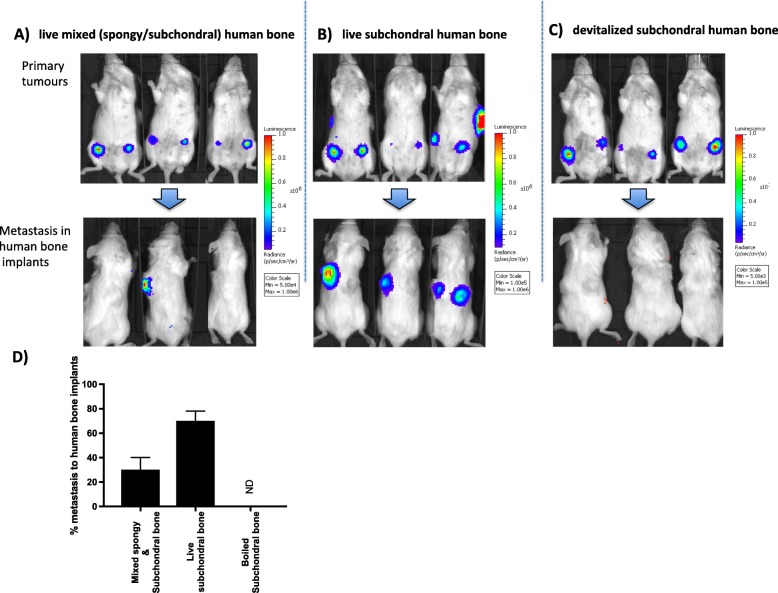
Effects of bone viability on breast cancer metastasis. Eight-week-old female NOD/SCID mice were implanted with femoral bone (mixed, spongy and subchondral) (**a**), subchondral only (**b**) or subchondral bone that had been devitalized prior to implantation (**c**). Four weeks after implantation of bone, all mice received injections of MDA-MB-231 breast cancer cells into mammary ducts 4 and 9; primary tumour growth and metastasis to human bone implants can be seen in **a** and **b**. Histogram (**d**) represents mean ± SEM of percentage of mice that developed metastases in human bone implants from 3 independent experiments each containing 10 mice/group, statistical significance determined by one-way ANOVA. **p < 0.001; ND, not detected

MDA-MB-231-TdTomato Luc2 breast cancer cells were injected orthotopically into the 4th mammary fat pads of NOD/SCID mice 4-weeks after implantation of human bone discs using the same protocol set out by Kupperwasser et al. [14]. The orthotropic engraftment resulted in tumour growth at the primary site in 90% of animals and spontaneous metastasis to human bone in 20% (data not shown). Changing the tumour implantation method to intra-ductal tumour cell injection, where breast cancer cells were injected via the 4th nipples into the mammary ducts [19] increased percentage of spontaneous metastasis to 30% (Fig. 4a). We have previously shown that sub-chondral human femoral bone is more metabolically active than spongy bone [20]; when only sub-chondral bone was used, metastasis to these implants further increased to 70% (Fig. 4b). MDA-MB-231-TdTomato Luc2 cells specifically metastasised to live human bone implants; when bone was de-vitalised by boiling before implantation, tumour cells did not metastasise to this site (Fig. 4c, d) and no metastasis to mouse organs were observed. We therefore carried out all future experiments using sub-chondral bone to mimic the human bone metastatic site, and tumour cells were administered via intra-ductal injection.

The majority of breast cancers that metastasise to bone are ER+, and in vivo models that represent this condition are lacking. We therefore tested the ability of ER+ MCF7 and T47D cells to metastasise to human bone implants. In NOD/SCID mice, we were unable to grow MCF7 or T47D cells in the mammary ducts without oestrogen supplementation (data not shown). Oestrogen has bone anabolic effects that have been shown to increase the metastatic potential of ER+ breast cancer cell lines [17]. To minimise these effects, mice were supplemented with the lowest dose of oestradiol that we found to be sufficient to support growth of the primary tumour, 12 mg/L. Using this dose of oestradiol resulted in primary tumour growth in 80% of mice injected with T47D cells and 70% of mice injected with MCF7 cells; however, no metastases were detected from either of these cell lines 5 weeks after injection. Following excision of the primary tumours, metastases developed in the human bone implants (50–60% of animals) and in mouse bone (40–50%) over an 8-week time period (data for T47D cells shown in Additional file 1: Figure S1).

### Metastasis of PDXs to human bone implants

Having established that human breast cancer cell lines preferentially metastasise to a human-specific site (human bone implant), we then developed PDX models. We hypothesised that breast cancer PDXs may also metastasise to this site, providing us with a model that more closely resembles human disease. A selection of PDXs (ER− PR− HER2−, ER+, PR+ HER2−, ER+ PR− HER2− and ER− PR− HER2−) that have never been cultured in vitro but have been propagated in NOD/SCIDy mice were used to increase the likelihood of tumour take (Table 1) [18]. To model spontaneous metastasis, tumours were injected into the 4th mammary ducts, and to mimic direct seeding from the blood, tumour cells were injected into the left cardiac ventricle 4 weeks after implantation of human bone. Intra-ductal injection of PDXs resulted in metastasis to human bone, mouse bone, and the lung with different frequencies depending on tumour type (Table 2). ER+ PDXs that metastasised to bone (BB3RC32 and BB2RC08) preferentially metastasised to human bone over mouse bone (100% vs. 75%; 100% vs. 20% respectively) whereas triple-negative PDXs preferentially metastasised to the lung compared with bone (100% vs. 20% mouse and 20% human).

**Table 1:** Summary of PDX models and metastatic potential. PDX breast cancer sub-types and metastasis status of patients from which cells were isolated

Model name	Pathology	Type	Grade	PDX model creation	Metastasis status/site in patient
BB3RC31	IDC (PE)	Late breast cancer	2	Cells isolated from pleural effusion	Lung, liver and peritoneum at time of drain
BB3RC32	IDC (Asc)	Late breast cancer	2	Cells isolated from ascetic fluid sample	Liver, lung and peritoneum at time of drain.
BB2RC08	IDC	Late breast cancer	Unknown	Created from primary breast tumour	No metastasis or recurrence to date.
BB6RC39	IDC	Early breast cancer	2	Created from primary breast tumour	No metastasis or recurrence to date.
BB6RC37	IDC	Early breast cancer	3	Created from primary breast tumour	No metastasis at time of PDX generation. Patient since diagnosed with metastasis to bone, liver and peri-pancreatic node.
BB6RC52	IDC	Early breast cancer	3	Created from primary breast tumour	No metastasis at time of PDX generation. Patient since diagnosed with brain metastasis.

**Table 2:** Summary of PDX models and metastatic potential. PDX take rate and sites of metastasis following intraductal injection into NOD SCID mice

Model name	Receptor status	Primary tumour growth	Metastasis from primary tumour			Metastases from intra-cardiac injection		
			Mouse bone	Human bone	Lung	Mouse bone	Human bone	Lung
BB3RC31	ER+ PR+ HER2−	100%	0%	0%	60%	NT	NT	NT
BB3RC32	ER+ PR+ HER2−	80%	75%	100%	70%	80%	80%	40%
BB2RC08	ER+ PR+ HER2−	100%	20%	100%	60%	40%	80%	80%
BB6RC39	ER+ PR+ HER2+	0%	0%	0%	0%	NT	NT	NT
BB6RC37	ER− PR− HER2−	100%	20%	20%	100%	30%	30%	100%
BB6RC52	ER− PR− HER2−	40%	0%	0%	0%	NT	NT	NT

Seeding PDX cells directly into the blood slightly reduced metastases to human bone in ER+ PDXs (100 to 80%, in mice injected with BB3RC32 or BB2RC08 cells via intra-ductal injection compared with intra-cardiac) and increased metastasis in triple-negative BB6RC37 from 20 to 30%. However, intra-cardiac injection of PDXs consistently increased metastases in mouse bone (Table 2).

**Fig. 5:**
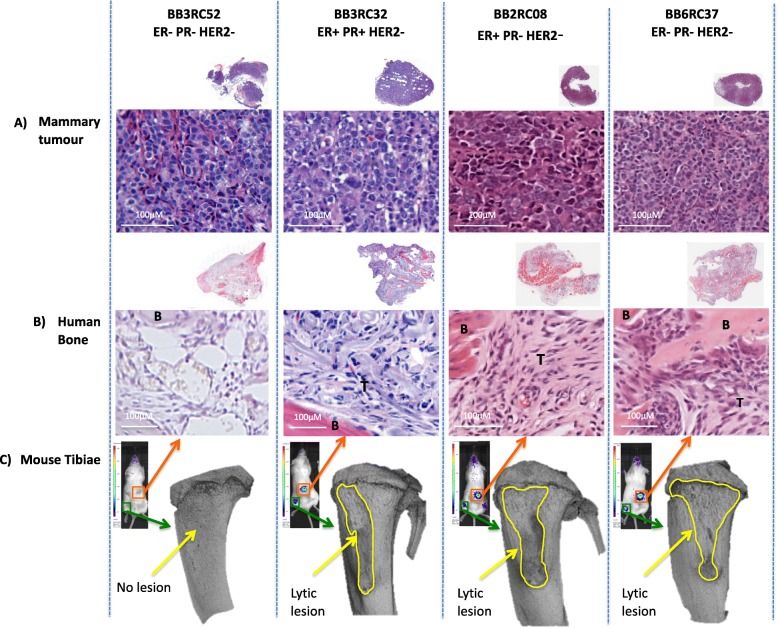
Effects of intraductal injection of breast cancer PDX on metastasis to human bone implants. Intraductal injection of a sub-set of breast cancer PDX into NOD/SCID mice pre-implanted with human subchondral bone results in metastasis to human/mouse bone. Representative photomicrographs (× 60) of primary tumour sections and human bone sections are shown in (**a** and **b** respectively) and uCT images of mouse tibiae are shown in (**c**). Human bone metastases in mice from which representative H&E images are shown are outlined in orange squares. Metastases in mouse bone from which uCT images are shown are highlighted in green squares, and lesions in mouse tibiae are outlined in yellow

Histological examination of the tumours revealed phenotypical differences between tumour cells growing at the primary site and those growing in the human bone disc. Irrespective of their sub-type, PDXs growing in the primary site are epithelial in appearance whereas in the human bone these same cells take on a fibroblastic appearance (Fig. 5), suggesting that cells undergo a distinct set of molecular changes that enable them to grow in this altered metastatic environment. These molecular changes did not result in altered hormone receptor status, all tumours displayed the same ER/PR/HER2 phenotype in both primary and metastatic sites and these were representative of the phenotypes described when the tumours were originally isolated from patients (Additional file 2: Figure S2) [18]. All PDXs that formed metastases in mouse bones produced lytic lesions (Fig. 5). Patients with breast cancer bone metastases develop lytic lesions; therefore, our data suggest that PDXs behave in a similar manner in mouse and in humans.

### Cancer cells undergo molecular changes associated with bone metastasis

**Fig. 6:**
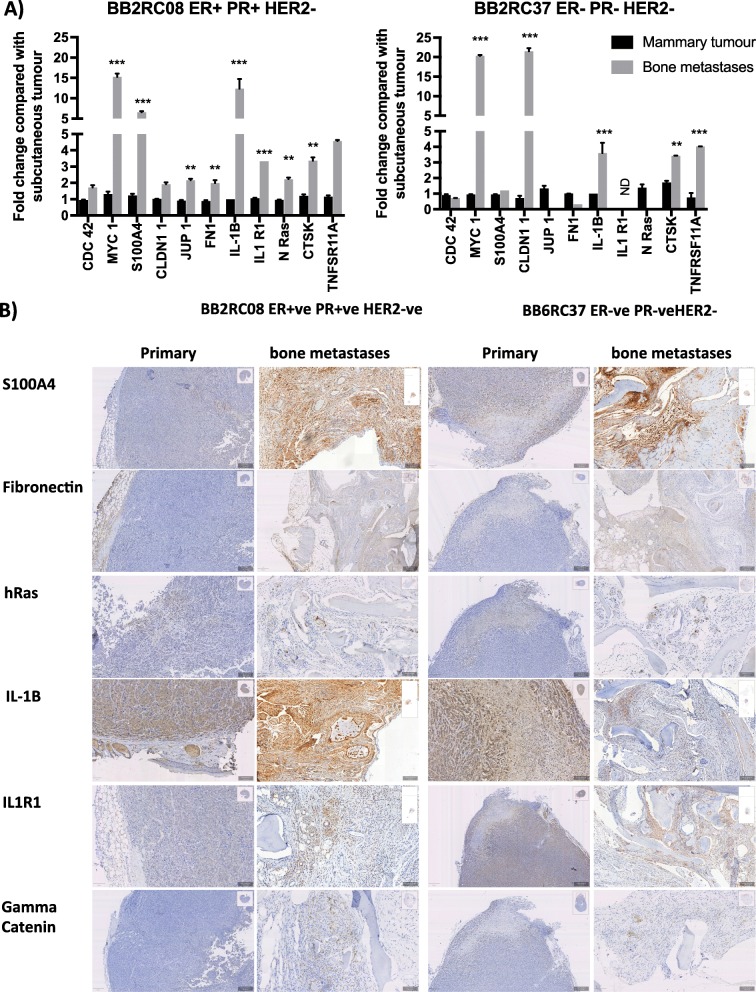
Gene and protein expression in primary tumours and matched bone metastases from ER+ and ER− PDX**. a** Fold change in gene expression from RNA isolated from human bone metastatic deposits compared with the corresponding primary PDXs. Data shown are mean ± SEM for 2 independent repeats of 3 replicates. **p < 0.001, ***p < 0.0001. **b** Photomicrographs of primary tumours and metastases in human bone implants following immunohistochemical detection of S100A4, Fibronectin, Ras IL-1B, IL1R1 and γ Catenin. All data shown are from ER+ PR+ HER2− BB2RC08 and ER− PR− HER2− BB2RC37 PDXs

Research into molecular mechanisms that drive breast cancer bone metastases has been hampered by lack of available clinical material from metastatic sites for study. Bone biopsies are rarely taken from patients with breast cancer, and the quality of this material is often poor resulting in there being few matching pairs of primary breast tumour and the corresponding bone metastases being available for research. Therefore, we used our humanised models of breast cancer metastasis to determine the molecular changes that occur in tumours during the different stages of metastasis. Whole genome Affymetrix arrays identified 50 genes that were altered in expression between MDA-Td Tomato tumours growing in the mammary duct that subsequently metastasised to human bone implants, compared to those that did not metastasise (Additional file 4: Table S1). When metastatic mammary MDA-Td Tomato tumours were compared with their resulting bone metastasis and naïve human bone, 1261 genes were altered in expression. Importantly, the gene expression profile of naïve bone implants, isolated from the same donor, was excluded from the equation before downstream analysis. Analysis using DAVID pathway mapping software identified 11 primary pathways associated with bone metastasis, with cytokine-cytokine receptor interactions being the most prominent pathway altered between tumours growing at the primary site and their resulting metastatic deposits (Additional file 3: Figure S3). The expression profile of genes that are central to one or more of these pathways were assessed by real-time quantitative PCR in MDA-Td and T47D cells grown in vitro, isolated from mammary tumours that did not metastasise, mammary tumours that did metastasise, circulating tumour cells and metastases isolated from human bone implants (Tables 3 and 4). The expression profile of genes altered between primary tumours and bone metastasis were further investigated in a triple-negative (BB2RC32) and an ER+ (BB2RC08) PDX model of metastasis to human bone, to identify common genes whose expression are altered as tumour cells move from the primary site to bone (Fig. 6). Using this method of selection, we identified 5 genes that are central to pathways involved in metastasis that were changed in all triple-negative and ER+ breast cancers tested, *MYC*, *CLDN1*, *IL-1B*, *CTSK* and *TNFRSF11A.* 
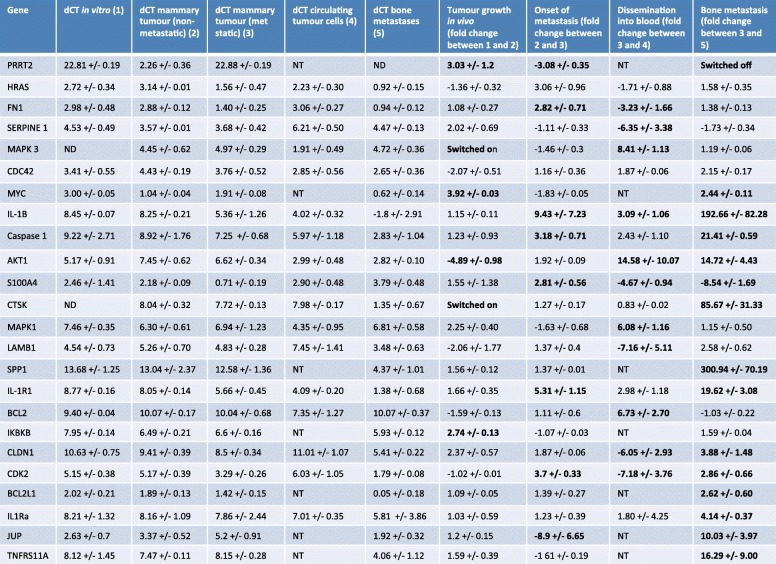

**Table 3:**
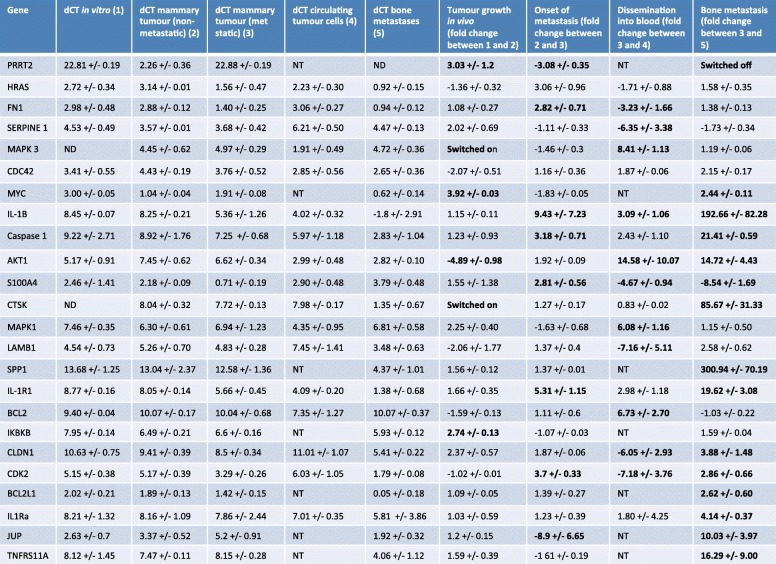
Genetic changes incurred during different stages of breast cancer metastasis to bone in triple-negative MDA-MB-231 cells. *ND* not detectable, *switched on* only detected in the comparison group, *NT* not tested


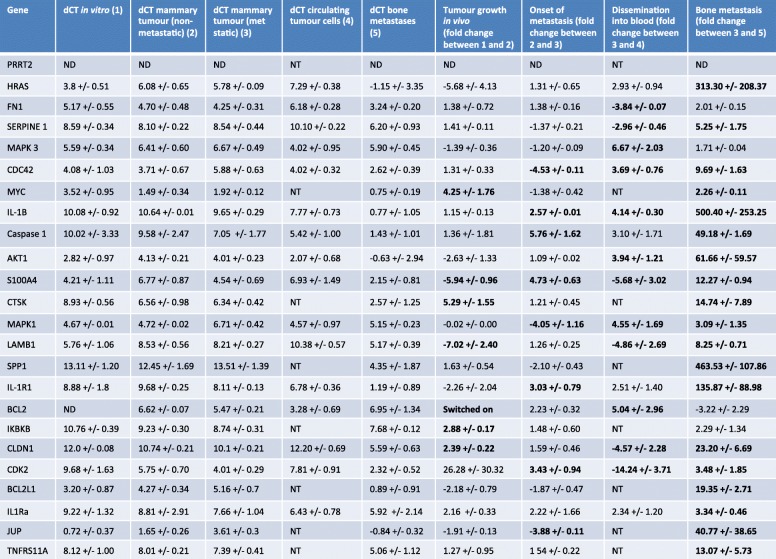

**Table 4:**
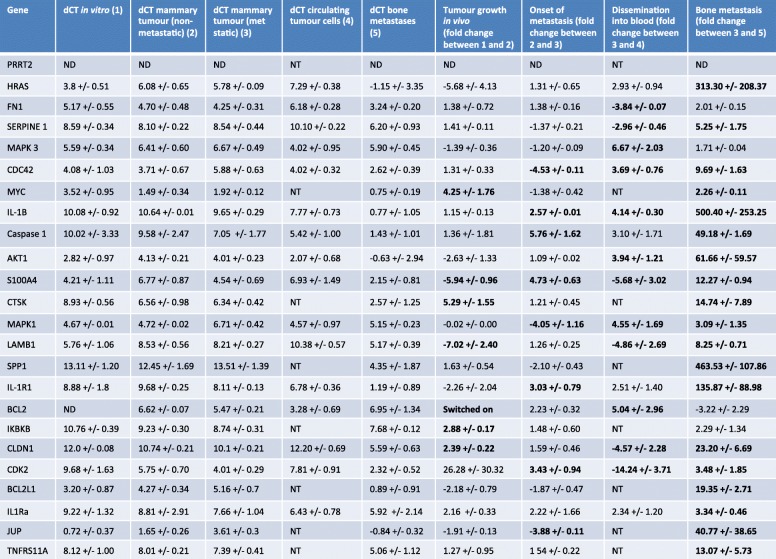
Genetic changes incurred during different stages of breast cancer metastasis to bone in ER+ T74D cells. *ND* not detectable, *switched on* only detected in the comparison group, *NT* not tested

We next established if changes in gene expression correlated with altered protein expression. Immunohistochemistry for S100A4, Fibronectin, RAS, IL-1B, IL-1R1 and γ Catenin was performed on mammary tumours and metastases in human bone implants. These selected proteins were chosen because they were predicted to be altered in either ER+ BB2RC08, triple-negative BB6RC37 or both of these PDXs and have previously been associated with bone metastasis [10, 11]. The calcium-binding protein S100A4 and cell adhesion protein fibronectin were both expressed at low levels in primary mammary tumours and increased in bone metastasis. The small GTPase RAS appeared to be unaltered between primary tumours and bone metastases in BB2RC08 cells and marginally decreased in BB6RC32 cells. Interestingly, the pro-inflammatory cytokine IL-1B and its receptor IL1R1 were increased in bone metastases compared with primary tumours in ER+ BB2RC08 cells but appeared to decrease in ER-ve BB6RC37 cells, and similar pattern was observed with the cell-cell adhesion molecule γ Catenin. Taken together, our data suggest that in our humanised model of breast cancer bone metastasis tumour cells undergo specific molecular alterations as they progress through different stages of the metastatic cascade.

### IL-1 signalling is important driver of bone metastasis

**Fig. 7:**
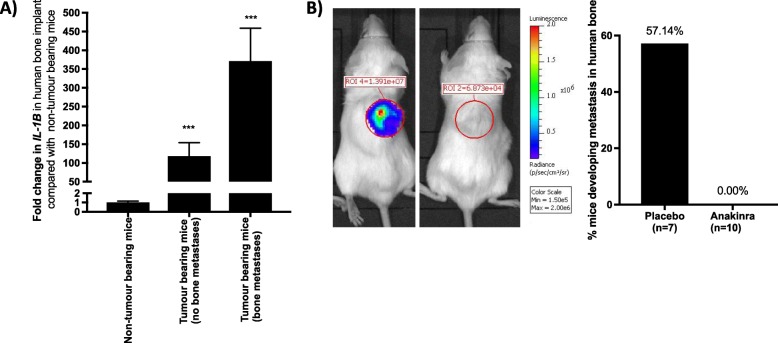
IL-1B signalling drives breast cancer metastasis to bone. **a** Gene expression of IL-1B in human bone implants isolated from NOD SCID mice 4 weeks after intraductal injection of MDA-Td Tomato cells. Data shown are from human bone implants bones isolated from mice in which MDA-Td Tomato cells did not metastasise and from human bone implants with MDA-Td Tomato metastases compared with bone from the same patient isolated from a mouse not injected with breast cancer cells. Data shown are mean ± SEM for *n* = 5–10 mice per group. Statistical significance determined by one-way ANOVA, *p < 0.01, **p < 0.001, ***p < 0.0001. **b** Effects of inhibiting IL-1 signalling on metastasis to human bone implants. MDA Td Tomato cells were othrotopically injected into NOD SCID ϒ mice 4 weeks after implantation of human bone. Mice were randomised to receive 1 mg/kg/day anakinra or placebo. Percentage of animals with metastasis in human bone was quantified 8 weeks post tumour cell injection. Data shown are from 7 to 10 mice per group

We have previously demonstrated that IL-1B in primary tumours correlates with future relapse in distal organs including bone in breast cancer patients [10] and have now demonstrated that IL-1B is altered between primary tumours and bone metastasis in cell lines and PDXs (Tables 3 and 4 and Fig. 6). We, therefore, investigated the importance of IL-1 signalling in promoting bone metastasis in our humanised models. Affymetrix array and real-time PCR analysis reviled significantly increased IL-1B in human bone discs implanted into mice 4-weeks after intra-mammary injection of tumour cells into the mammary ducts compared to human bone discs isolated from mice that did not receive injections of tumour cells. Il-1B increases 118 +/− 36 fold in human bone isolated from mice injected with MDA-Td Tomato cells that did not metastasise and 371+/− 88 fold in bone isolated from mice injected with MDA-Td Tomato cells that did metastasise compared with bone isolated from mice that were not injected with tumour cells (Fig. 7a). Treating mice with 1 mg/kg/day of the IL-1Ra anakinra reduced numbers of mice that developed metastasis in human bone implants from (57.14%) to (0%) (Fig. 7b). These data highlight the importance of IL-1 signalling in development of bone metastasis.

In conclusion, our models provide valuable pre-clinical tools to study breast cancer bone metastasis and identify mechanisms driving this disease in patients. This model can be used to test pharmacological approaches to stop breast cancer metastasis, allowing transition from bench to bedside.

## Discussion

Modelling human breast cancer metastasis to bone for the development of future therapies relies on recapitulating every step in the metastatic cascade, as well as incorporating a species-specific microenvironment for secondary tumour colonisation. In the current study, we have developed the first PDX models of ER+ and ER- breast cancers that grow at the primary site and spontaneously metastasise to a human bone environment. Breast cancer cells utilised in these models undergo clinically relevant molecular changes as they progress through the metastatic process. Furthermore, molecules identified as metastatic drivers in this model can be used to predict future relapse in bone in breast cancer patients [10], highlighting the potential impact of this humanised model system in delivering translatable data from the laboratory to the clinic.

Researchers have previously attempted to develop mouse models, such as the humanised model described here, to represent human breast cancer metastasis to a human bone environment. However, low rates of metastasis and the use of just one triple-negative breast cancer cell line (SUM1315) [14, 21] suggest underlying problems with this approach. In an attempt to increase rates of metastasis, researchers modelling prostate cancer have used foetal bone [16, 21]. This bone differs significantly in composition to adult bone including large numbers of stromal cells and an absence of plasma cells [22]. Moreover, there are ethical considerations using this material, and metastasis rates remain low. These difficulties have led to exploration of using artificial scaffolds to mimic the metastatic bone environment [23–25]. Our previous research and research performed by other laboratories have established the importance of bone-bound cytokines and growth factors in bone metastasis, as well as the need for viable, metabolically active bone cells (osteoclasts, osteoblasts and osteocytes) to facilitate bone turnover and promote the vicious cycle of cancer-induced bone disease [20, 26]. Artificial scaffolds do not fully represent the human bone environment: the lack of human-specific growth factors bound within the bone matrix and limited representation of cell types found in bone do not allow complete recapitulation of tumour cell-bone cell interactions that occur during the metastatic process [20]. We have therefore focused our efforts on developing a reliable mouse model of human breast cancer metastasis to human bone, in which ER+ and ER-ve breast cancer cell lines and PDXs spontaneously metastasise to adult human bone implants with high efficiency and reproducibility.

Architectural and cellular composition of trabecular bone varies between patients and is dependent upon a number of factors, including direct mechanical stimuli but also age and sex of individuals, nutrition, co-morbidities, use of medication and the level of physical activities that affect bone metabolism [27]. For the current study, we, therefore, selected to use bones from post-menopausal women (average age 63, without previous exposure to anti-resorptive drugs) undergoing total hip replacement surgery as a result of rheumatoid arthritis, having previously shown that variability in trabecular bone content is low in these patients [20]. As we are achieving consistent rates of metastasis to bone implants in our cell line models of between 60 and 80% (Fig. 4d), bones from these donors appear to be an appropriate tissue for generating a human-specific metastatic site.

Recently, understanding the role of the bone metastatic niche for cancer cell homing to this organ has become important. It is thought that metastatic cancer cells occupy a similar/partly overlapping niche to haematopoietic stem cells (HSCs) [28, 29]. Recent studies investigating the regional localisation of HSCs within the bone marrow has identified their preference for endosteal regions [29, 30] due to the high cellular composition of osteoblasts in particular. We hypothesise that cancer cells may also be attracted to similar osteotropic signals and have demonstrated that mobilisation of HSCs from the niche to the circulation prior to injection of breast cancer cell lines results in increased numbers of tumour cells disseminated in bone [29]. Maintenance of an osteotropic phenotype following bone core xenotransplantation may, therefore, be a key strength of this model. Examination of human B cells from the implanted human bone marrow migrating to the mouse spleen indicates the functionality of the bone marrow as well as the appropriate transfer of cells between organs. Engraftment must therefore involve revascularisation of the bone cores, a process that is tissue-specific. In primary patient tumour xenografts, the persistence of human vessels supporting their growth is generally poor, with engraftment and growth associated with recruitment and incorporation of the host vasculature [31], with time frames of host vessel growth differing between tumour type. Human skin grafts also demonstrated the gradual replacement of human vessels by murine vessels [32]. Revascularisation of human bone in breast cancer models has been shown to involve vascular endothelium of both xenograft and host species origin [14, 15], although the proportions were not quantified. By dual staining the vascular endothelium, this study demonstrated that human immunogenic stimuli persisted in the bone cores. As the total number of vessels in the bone implants did not change over time, but vessels were lined with a mixture of human and mouse endothelial cells, this supports a mechanism of repopulation of existing human vessels by the host, rather than new mouse vessel formation. This is the first time this has been shown in adult bone grafts.

Revascularisation of the bone implant represents another variable in the efficiency of tumour colonisation in this model, and species origin may contribute to the initial colonisation, establishment and growth of metastatic colonies in human bone [21]. For example, in vitro studies have identified a number of molecules for the preferential adhesion of human prostate cancer [33, 34] and breast cancer cells [35] to human endothelial cells. These tissue-specific interactions have also been demonstrated by the higher tumorigenicity observed in breast cancer cells when implanted into human breast tissue at the orthotopic site [36]. This may also be true for components of the bone, including the bone cells (osteoblasts and osteoclasts) and the proteins they secrete as well as the physical properties of the bone.

In our humanised model, we found a reduction in the numbers of human bone cells (osteoclasts, osteoblasts and osteocytes) in the human bone 7–14 days after implantation, indicating an initial loss of viability (Fig. 1). In this early period, blood vessels were also undergoing graft-related changes and were not functioning or functioning at low levels as indicated by the inability to traffic human B cells from the bone implant to the spleen 7 days following bone engraftment (Figs. 2 and 3). By week 4 following implantation, numbers of osteoclasts and osteoblasts had returned to levels comparable to baseline, new bone was actively being laid down and B cells were being successfully transported out of the human bone through the circulation. Interestingly, by this time point, the majority of cells involved in bone turnover (osteoclasts and osteoblasts) were of mouse origin, indicating that the resulting bone graft is a chimeric tissue comprised of human bone matrix complete with human-specific bone-bound growth factors that is being remodelled by mouse osteoclasts and osteoblasts. The impact of increasing ‘murinisation’ of the human bone implant over time remains to be established. Affymetrix array analysis of naïve (non-tumour bearing) human bone implanted into NOD/SCID mice for 16 weeks detected > 1000 human-specific genes (data not shown), indicating that these implants retain human-specific RNA for long periods of time. Human breast cancer cell lines and PDXs also preferentially metastasised to human bone over mouse bone. Preferential metastasis to this site was not the result of inflammation or cytokine release during wound healing caused by implantation surgery, as tumour cells were only capable of metastasising to live human bone implants (Fig. 4). Taken together, our data suggest that human bone implants retain species-specific properties that attract human cancer cells. These tumour cell-attracting factors are likely to be bound in the bone matrix as active bone turnover is required for metastasis to occur, but the species from which the bone remodelling cells originate is not critical.

The importance of bone remodelling in the development of bone metastases is further highlighted in the ER+ and PDX models. Cells metastasised to human bone implants but also formed metastases (although to a lesser extent) in mouse bone. In order to stimulate growth of the primary tumours, mice received 17β oestradiol supplementation. Oestradiol has potent bone anabolic properties causing increased oestoblastogenesis and a resulting increase in osteoclast activity [17]. We have previously shown that increasing bone cell activity caused by oestradiol supplementation stimulates metastases of MCF7 and T47D cells in mouse bone that can be inhibited by the anti-resorptive agent zoledronic acid [17]. We therefore hypothesise that metastasis to mouse bone observed in our models is the result of the oestradiol-mediated increase in bone cell activity. In the current study, we were unable to test this hypothesis, as we were unable to grow MCF7 or T47D in the mammary ducts of NOD/SCID mice. We have previously shown that both of these cell lines grow following orthotopic injection into BALB/c nude mice in the absence of oestradiol supplementation, and Sflomos et al. have demonstrated increased growth of ER+ve cells following intraductal compared with orthotopic injection in SCID/beige and NOD/SCID Gamma mice [17, 37]. The mammary ducts in some strains of mice may therefore be more permissive for the growth of ER+ve breast tumours, than others. It would be useful to test this in more detail before further refining our ER+ve breast cancer bone metastasis models. Metastasis to the human bone implant may not require the additional stimulus of oestradiol. Mice injected with MDA-MB-231 (in the current study) or SUM1315 used in previous studies did not receive oestradiol supplementation, and these cells lines specifically metastasised to human bone implants [14]. Furthermore, breast cancer cells all preferentially metastasised to human bone compared with mouse bone, emphasising the likelihood of species-specific factors driving human breast cancers to metastasise to human bone. The identification of these factors requires further investigation.

Unlike breast cancer cell lines, PDXs that metastasised from the primary site did not exclusively metastasise to bone. BB3RC32 and BB2RC08 preferentially metastasised to bone; however, all metastatic PDXs spread to the lungs and BB3RC31 and BB6RC37 preferentially metastasised to lungs compared with bone (Tables 1 and 2). This finding was not unexpected as the PDXs chosen for the current study had all previously been shown to develop lung metastasis from sub-cutaneous implants in NOD/SCID/γ mice [18]. Importantly, all PDXs retained their hormone receptor status throughout the experiment, PDXs that expressed ER, PR and/or HER2 in the original PDX continued to express these receptors in primary and metastatic tumours produced developed in this humanised model (Additional file 2: Figure S2).

Breast cancer bone metastasis is currently incurable. The ability to develop new and effective treatments is dependent on researchers gaining an in-depth understanding of how tumour cells progress through the metastatic process and how they interact with their host environment. We have demonstrated that both breast cancer cell lines and PDXs undergo significant genetic changes during this multistep process: Despite being a clonal cancer cell line, 50 genes were altered between MDA-MB-231 TdTomato Luc2 mammary tumours that spontaneously metastasised to human bone implants compared with mammary tumours that did not metastasise (Additional file 4: Table S1). A further 1217 genes were changed between metastatic mammary tumours (primary) and their corresponding metastatic deposits isolated from the human bone implants (Additional file 3: Figure S3C). Importantly, follow-up analysis by real-time PCR confirmed that a number of genes associated with osteomimicry (*CTSK*, *SPP1* and RANK) were upregulated in ER+ and triple-negative breast cancer cell lines and PDXs that had metastasised to bone, compared with their associated primary tumours [38]. In addition, proteins that have previously been associated with increased bone metastasis including the calcium-binding protein and fibroblast marker S100A4 as well as the cell adhesion molecule fibronectin [11] were both increased in bone metastases from ER+ and triple-negative PDXs compared with the primary tumour, and γ catenin, previously shown to be dysregulated in ER+ models of bone metastasis [39], was increased in the bone metastatic deposits from ER+ PDXs but not triple negative. These data support the phenotypical changes observed between PDXs appearing more epithelial-like when growing in the primary site but changing to a more fibroblastic appearance following metastasis to human bone implants (Fig. 5). Importantly, this model identified cytokine-cytokine receptor interactions as being principle drivers of bone metastasis. Our new data indicate that IL-1B is increased in both the primary and metastatic site prior to the onset of metastasis and blocking activity of the IL1R inhibits development of bone metastasis. The data are in line with our previously published research in which we identified IL-1B as being upregulated in mammary tumours that metastasised to human bone compared with mammary tumours that did not metastasise [10, 11]. Furthermore, analysis of IL-1B expression in tumour samples from > 1000 patients with stage II/III breast cancer revealed that patients whose primary tumours expressed IL-1B were significantly more likely to develop metastasis to bone (*p* = 0.017) or other organs (*p* = 0.0016) compared with patients whose tumours did not express IL-1B, over a 10-year follow-up period [10]. Whether increased IL-1B in the bone environment primes the bone metastatic niche and/or stimulates tumour cells to home to this organ remains to be established. Our previous data suggests that inhibiting IL-1 does not inhibit tumour cell homing to this environment, but prevents outgrowth of disseminated tumour cells in bone by inhibiting expansion of the bone metastatic niche [10, 40]. It therefore appears that increased IL-1B, observed in bone metastasis, may stimulate the niche leading to outgrowth tumour cells disseminated in this site. Taken together, these data suggest that in our humanised mouse model of bone metastasis, tumour cells that have metastasised to bone behave in a similar manner to those previously described for other model systems and human patients [10, 11, 41]. We are therefore confident that this model of human breast cancer metastasis to human bone implants represents a clinically relevant model for the future development and testing of new treatments for breast cancer bone metastasis.

## Conclusions

We have developed the first PDX models of ER+ and ER-ve breast cancers that grow at the primary site and spontaneously metastasise to a human bone environment with high frequency and reproducibility. Breast cancer cells utilised in these models undergo clinically relevant molecular changes as they progress through different stages of the metastatic process. This model can successfully identify metastatic drivers of breast cancer bone metastasis and may therefore represent a useful model for generating data that is translatable from the laboratory to the clinic.

## Supplementary information

**Additional file 1: Figure S1.** Spontaneous metastasis of T47D cells from mouse mammary ducts to human bone implants and mouse bone. Intra nipple injection of T47D cells into NOD SCID mice supplemented with 4 mg/L 17β oesteradiol results in tumour growth at the primary site. 8-weeks after resection of primary tumours metastases were detected in both human bone implants and mouse hind limbs.

**Additional file 2: Figure S2.** Confirmation of ER/PR/HER2 expression in PDX samples. Gene expression analysis of estrogen receptor (ESRRG), progesterone receptor (PGR) and HER2 (ERBB2) in PDX’s growing in the mammary gland and in metastatic deposits isolated from human bone implants. Histograms show mean delta CT +/− SD of the expression of gene of interest compared with the housekeeping gene GAPDH.

**Additional file 3: Figure S3.** Alterations in molecular pathways associated with bone metastasis. Heat map showing primary molecular pathways altered in MDA-MB-231 cells that have metastasised to human bone implants compared with the corresponding mammary tumours as assessed on whole genome Affymetrix arrays, analysed using DAVID (A). The number of genes altered between primary tumours that metastasised to bone compared with those that did not and the number of genes that changes between met static and non-met static primary tumours and bone metastases are shown in B. Panel C, shows the genetic pathways altered between primary tumours that metastasised to bone and metastatic deposits isolated from human bone implants.

**Additional file 4: Table S1.** Affymetrix array analysis showing genetic alterations between MDA-MB-231 cells that metastasise to human bone compared to cells that do not metastasise.

## Data Availability

Raw data files from Affymetrix arrays are available via the NCBI website (https://ncbi.nml.nih.gov) accession number GEO GSE137842. Tumour tissue from the humanised mouse models will be made available for use by other researchers through the NC3Rs SEARCHBreast initiative (http://Searchbreast.org) or through collaboration with Dr. Penelope Ottewell (University of Sheffield, UK). Data and metadata on PDX are available in PDX Finder (http://pdxfinder.org) and the EuroPDX data portal (http://dataportal.europdx.eu), and PDXs are available through the Breast Cancer Now biobank (www.breastcancertissuebank.org), or through direct collaboration with Prof Robert Clarke (University of Manchester, UK).

## Abbreviations

PDX: Patient-derived xenograft
ECACC: European Collection of Authenticated Cell Cultures
HTA: Human tissue act
OCT: Optimum cutting temperature compound
uCT: Micro-computed tomography
BV: Bone volume
TV: Tissue volume
TRAP: Tartrate-resistant acid phosphatase
PRRT2: Proline-rich transmembrane protein 2
HRAS: Harvey Rat Sarcoma Viral Oncogene
FN1: Fibronectin
SERPINE: Serine proteinase inhibitor 1
MAPK: Mitogen-activated protein kinase
CDC42: Cell division control protein 24 homologue
MYC: Myelocytomatosis proto oncogene
IL-1B: Interleukin 1beta
AKT1: Protein kinase B
LAMB1: Laminin 1
SPP1: Secreted phosphoprotein 1
IL1R1: Interleukin 1 receptor 1
BCL2: B cell lymphoma 2
IKBKB: I-kappa-B kinase beta
CLDN1: Claudin 1
CDK2: Cyclin-dependent kinase 2
BCL2L1: B cell lymphoma 2 like 1
IL1Ra: Interleukin 1 receptor antagonist
JUP: Junction plakoglobin
TNFSF11A: Tumour necrosis factor ligand superfamily number 11A
CTSK: Cathepsin K
GAPDH: Glyceraldehyde 3-phosphate dehydrogenase
VEGF: Vascular endothelial growth factor
RANKL: Receptor activator of nuclear factor kappa-B ligand
ACP5: Acid phosphatase 5, tartrate resistant
ALPL: Alkaline phosphatase
ER: Oestrogen receptor
PR: Progesterone receptor
HER2: Human epidermal growth factor receptor 2
S100A4: S100 calcium-binding protein A4
DKK: Dickkopf-related protein 1
FITC: Fluorescein isothiocyanate
TRITC: Rhodamine
SEM: Standard error of the mean
CTX: C-terminal telopeptide
HSC: Haematopoietic stem cell
