# Supplementary material for: Development of clinically relevant in vivo metastasis models using human bone discs and breast cancer patient-derived xenografts
Source: Breast Cancer Res. 2019 Nov 29;21:130. doi: 10.1186/s13058-019-1220-2 (PMC6884811; doi:10.1186/s13058-019-1220-2)

Figure S1

Intraductal injection of T47D cells into female NOD SCID mice 4-weeks after human bone graft

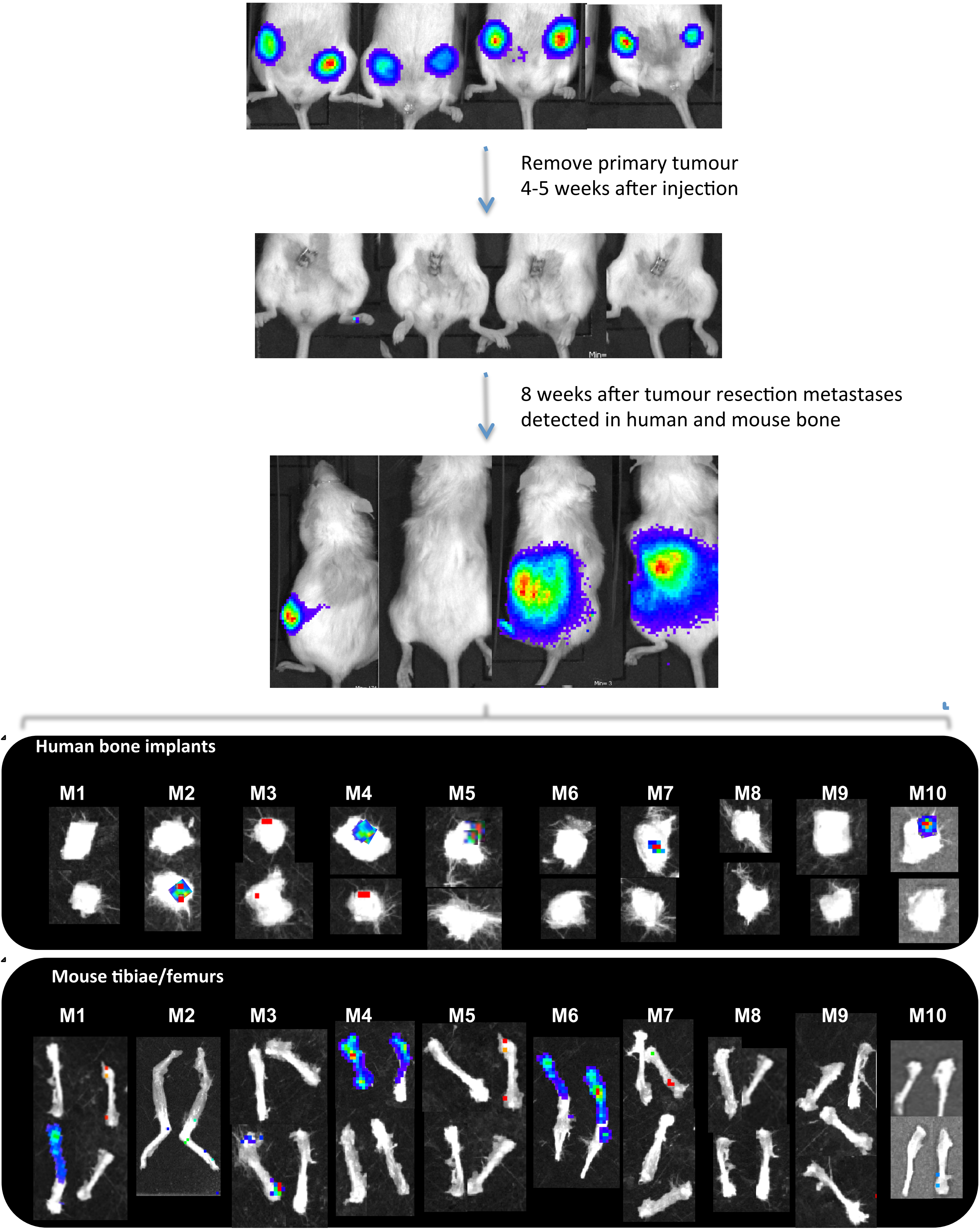

Supplement: Supplementary file 1 — Additional file 1: Figure S1. Spontaneous metastasis of T47D cells from mouse mammary ducts to human bone implants and mouse bone. Intra nipple injection of T47D cells into NOD SCID mice supplemented with 4 mg/L 17β oesteradiol results in tumour growth at the primary site. 8-weeks after resection of primary tumours metastases were detected in both human bone implants and mouse hind limbs. [file 13058_2019_1220_MOESM1_ESM.pdf]
