# Supplementary figures and images for: Development of clinically relevant in vivo metastasis models using human bone discs and breast cancer patient-derived xenografts
Source: Breast Cancer Res. 2019 Nov 29;21:130. doi: 10.1186/s13058-019-1220-2 (PMC6884811; doi:10.1186/s13058-019-1220-2)

Figure S2

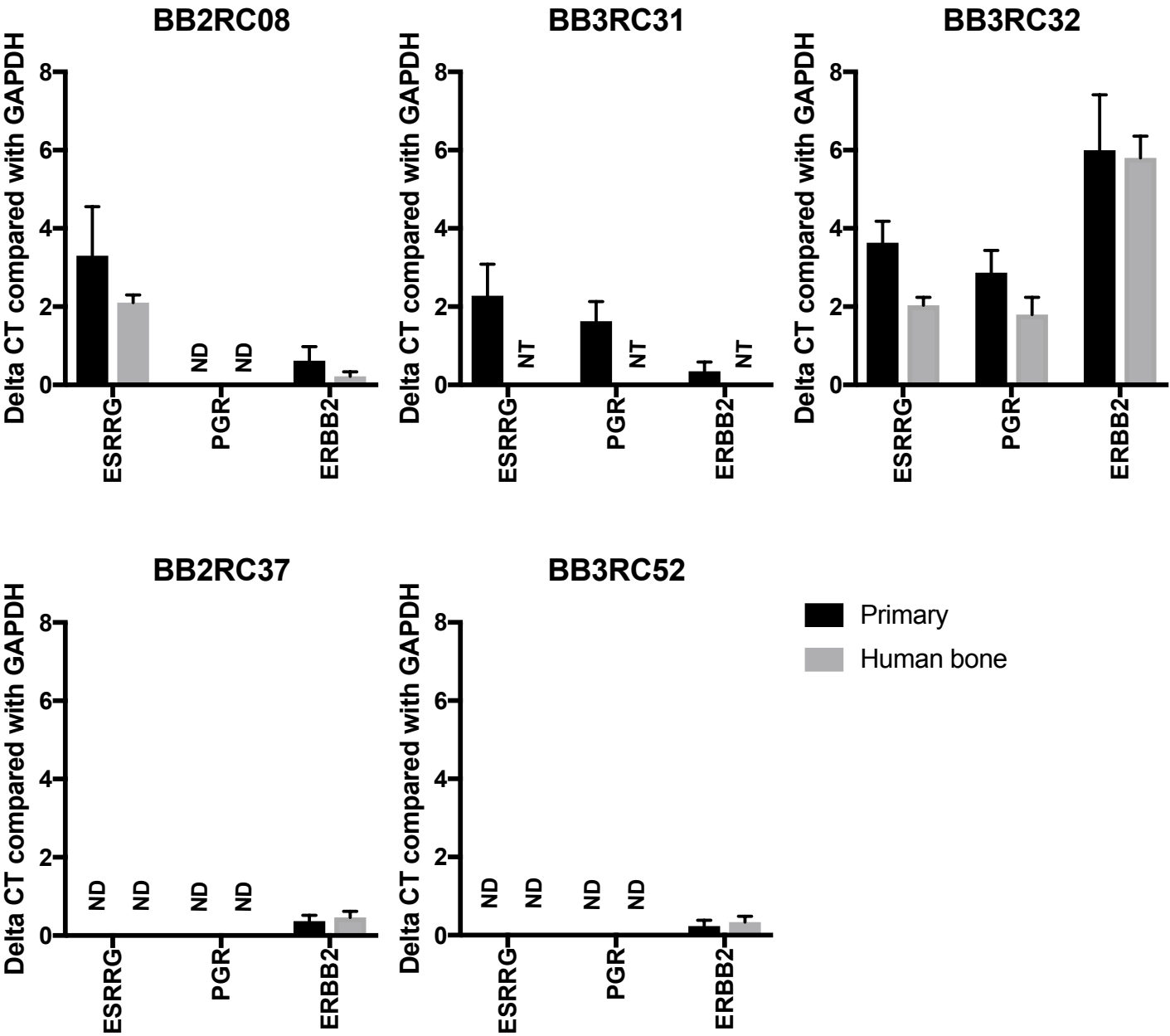

Supplement: Supplementary file 2 — Additional file 2: Figure S2. Confirmation of ER/PR/HER2 expression in PDX samples. Gene expression analysis of estrogen receptor (ESRRG), progesterone receptor (PGR) and HER2 (ERBB2) in PDX’s growing in the mammary gland and in metastatic deposits isolated from human bone implants. Histograms show mean delta CT +/− SD of the expression of gene of interest compared with the housekeeping gene GAPDH. [file 13058_2019_1220_MOESM2_ESM.pdf]

Figure S3

A)

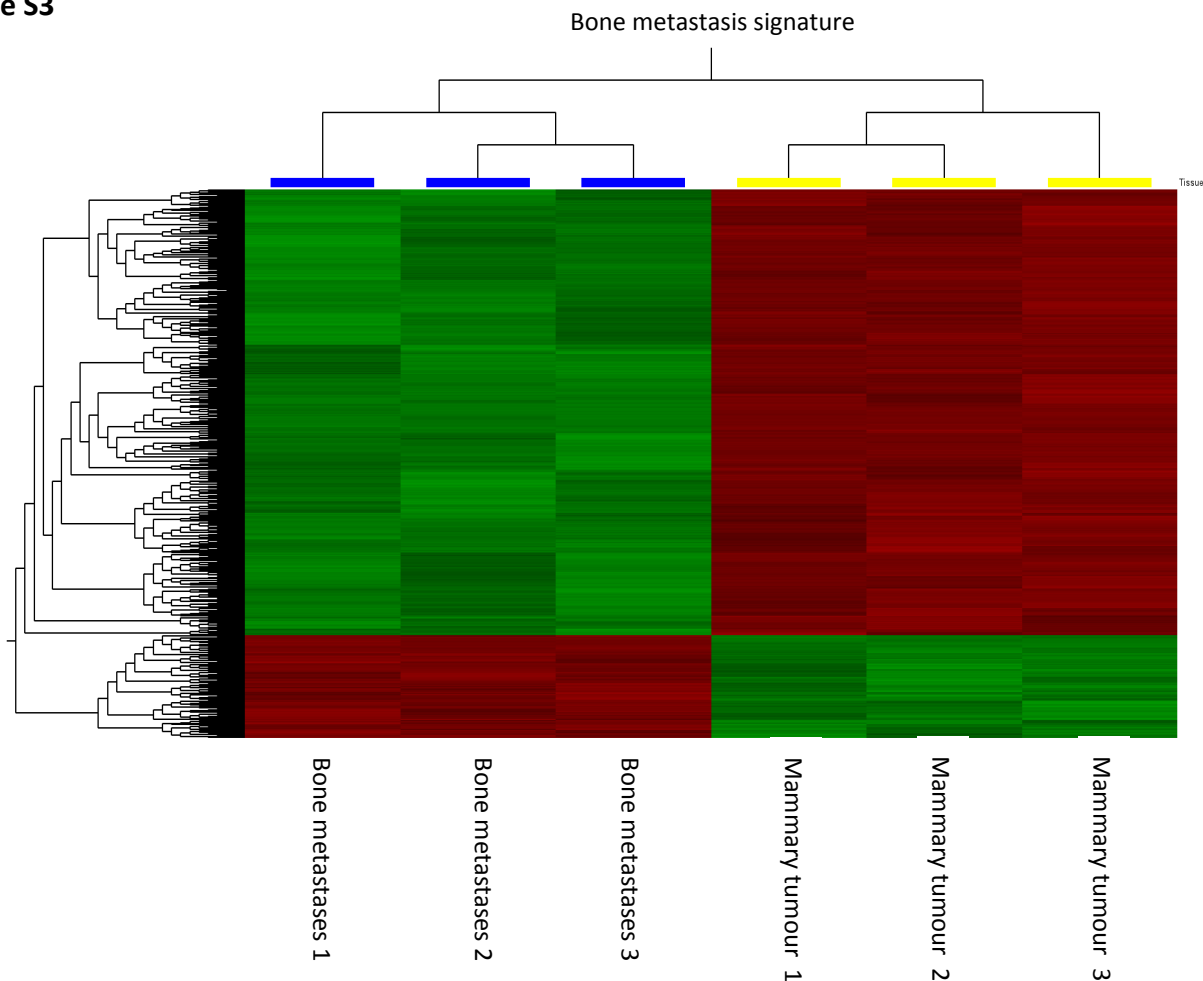

B)

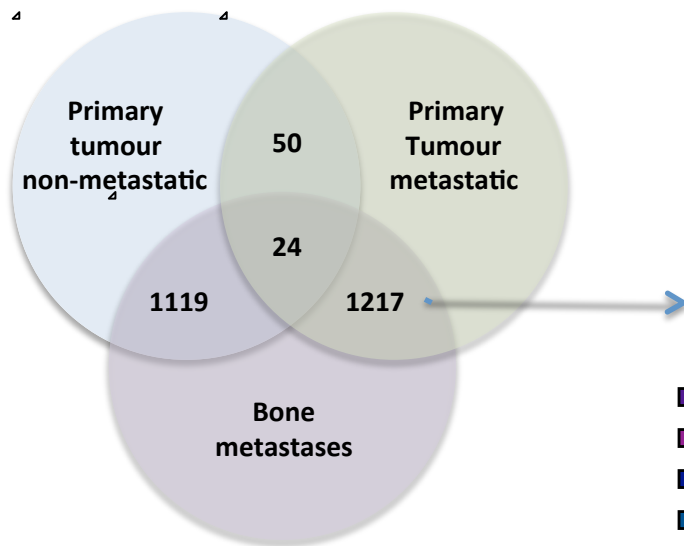

C)

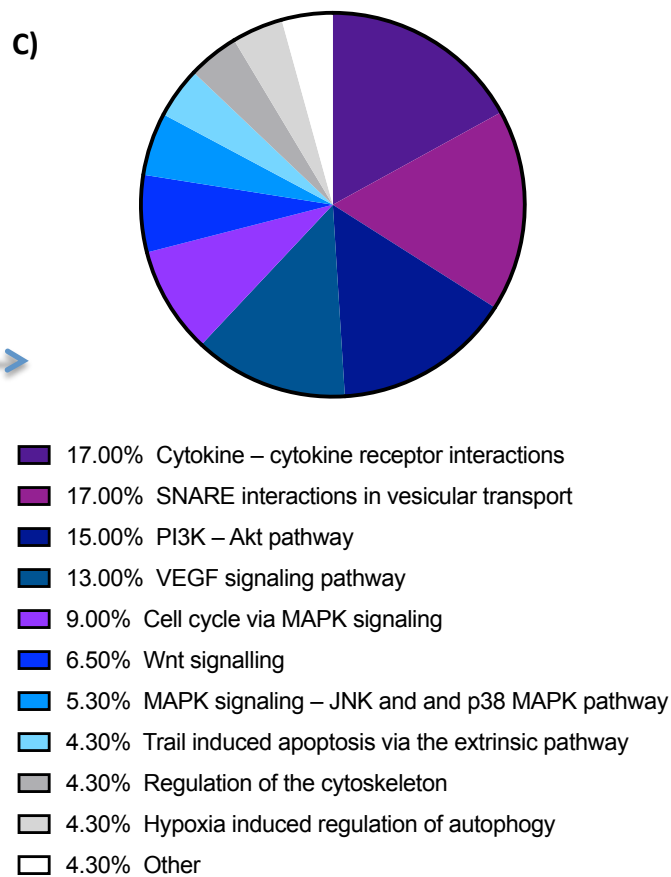

Supplement: Supplementary file 3 — Additional file 3: Figure S3. Alterations in molecular pathways associated with bone metastasis. Heat map showing primary molecular pathways altered in MDA-MB-231 cells that have metastasised to human bone implants compared with the corresponding mammary tumours as assessed on whole genome Affymetrix arrays, analysed using DAVID (A). The number of genes altered between primary tumours that metastasised to bone compared with those that did not and the number of genes that changes between met static and non-met static primary tumours and bone metastases are shown in B. Panel C, shows the genetic pathways altered between primary tumours that metastasised to bone and metastatic deposits isolated from human bone implants. [file 13058_2019_1220_MOESM3_ESM.pdf]
