# Supplementary material for: Development of clinically relevant in vivo metastasis models using human bone discs and breast cancer patient-derived xenografts
Source: Breast Cancer Res. 2019 Nov 29;21:130. doi: 10.1186/s13058-019-1220-2 (PMC6884811; doi:10.1186/s13058-019-1220-2)
Supplement: Supplementary file 4 — Additional file 4: Table S1. Affymetrix array analysis showing genetic alterations between MDA-MB-231 cells that metastasise to human bone compared to cells that do not metastasise. [file 13058_2019_1220_MOESM4_ESM.docx]

| **Gene Title** | **p-value** |
| --- | --- |
| Friend leukemia virus integration 1 | 1.17E-07 |
| zinc finger protein 99 | 1.53E-06 |
| chemokine-like receptor 1 | 3.14E-06 |
| dopamine receptor D2 | 4.63E-06 |
| endothelin receptor type B | 4.64E-06 |
| collagen, type I, alpha 2 | 5.13E-06 |
| uncharacterized LOC100507654 | 5.58E-06 |
| zinc finger protein 3 homolog (mouse) | 1.38E-05 |
| glycoprotein M6B | 2.10E-05 |
| Interleukin 1 beta | 2.55E-05 |
| ring finger protein 180 | 3.23E-05 |
| NAC alpha domain containing | 3.69E-05 |
| N-acetylated alpha-linked acidic dipeptidase 2 | 7.93E-05 |
| uncharacterized LOC100505782 | 7.97E-05 |
| Uncharacterized FLJ40288 | 8.13E-05 |
| major histocompatibility complex, class I-related | 0.000144236 |
| interleukin 1 receptor, type I | 0.00020234 |
| transition protein 1 (during histone to protamine replacement) | 0.00024102 |
| fibroblast growth factor 1 (acidic) | 0.000252479 |
| maternally expressed 3 (non-protein coding) | 0.00025668 |
| collagen, type V, alpha 1 | 0.000358084 |
| collagen, type I, alpha 2 | 0.000365524 |
| kelch domain containing 8A | 0.000465614 |
| leucine-rich repeats and immunoglobulin-like domains 1 | 0.000467317 |
| actin binding LIM protein 1 | 0.00056891 |
| sema domain, immunoglobulin domain (Ig), short basic domain, secreted, (semaphorin) 3D | 0.000688124 |
| family with sequence similarity 86, member A pseudogene | 0.000879897 |
| lysine (K)-specific demethylase 4B | 0.00091155 |
| collagen, type XVI, alpha 1 | 0.000998372 |
| proline-rich transmembrane protein 2 | 0.001156906 |
| S100 calcium binding protein A4 | 0.001310655 |
| leucine zipper, putative tumor suppressor 1 | 0.001495439 |
| potassium voltage-gated channel, Shaw-related subfamily, member 2 | 0.001834427 |
| caspase 1, apoptosis-related cysteine peptidase | 0.001938798 |
| schlafen family member 13 | 0.002293532 |
| protein phosphatase 1, regulatory subunit 12C | 0.002563538 |
| MCF.2 cell line derived transforming sequence | 0.004121989 |
| protein arginine methyltransferase 1 | 0.004814042 |
| SRY (sex determining region Y)-box 17 | 0.004865128 |
| cyclin-dependent kinase 2 | 0.008952807 |
| Fibronectin 1 | 0.014913702 |
| insulin-like 5 | 0.01520382 |
| uncharacterized LOC100507240 | 0.017195806 |
| centrosomal protein 192kDa pseudogene | 0.021484048 |
| RELT-like 1 | 0.022505914 |
| calcium binding protein 1 | 0.027401239 |
| actin-like 9 | 0.031748004 |
| carbonic anhydrase XII | 0.033421459 |
| cannabinoid receptor interacting protein 1 | 0.039799528 |
